# When Confidence in Institutions Backfires: Power‐Distance Orientation Moderates the Relationship Between Institutional Trust and Civic Honesty Across Eight Countries

**DOI:** 10.1002/ijop.70059

**Published:** 2025-05-28

**Authors:** Silvana D'Ottone, Giovanni A. Travaglino, Pascal Burgmer, Isabella Giammusso, Hirotaka Imada, Yanhui Mao, Alberto Mirisola, Chanki Moon, Kengo Nawata, Miki Ozeki

**Affiliations:** ^1^ School of Psychology Pontificia Universidad Católica de Chile Santiago Chile; ^2^ Institute for the Study of Power, Crime and Society, Department of Law and Criminology Royal Holloway University of London Egham UK; ^3^ School of Psychology University of Southampton Southampton UK; ^4^ Department of Psychology, Educational Science and Human Movement University of Palermo Palermo Italy; ^5^ Department of Psychology Royal Holloway University of London Egham UK; ^6^ Institute of Applied Psychology, Psychological Research and Counseling Center Southwest Jiaotong University Chengdu China; ^7^ Department of Psychology Jeonbuk National University Jeonju South Korea; ^8^ Faculty of Humanities Fukuoka University Fukuoka Japan; ^9^ Faculty of Humanities and Social Sciences Okayama University Okayama Japan

**Keywords:** civic honesty, confidence in institutions, corruption, power‐distance orientation

## Abstract

Confidence in institutions is a key predictor of civic honesty, yet evidence shows that this relationship varies across contexts and individuals. This study examined whether power‐distance orientation (PDO)—the extent to which individuals accept hierarchical power relations—moderates this association. High‐PDO individuals tend to view institutional authorities as entitled to privilege, inclined to engage in patronage relationships and potentially corrupt. We hypothesised that for individuals high in PDO, confidence in institutions could backfire and be linked to the rejection of civic honesty. Using data from 2088 participants across eight countries, we found support for this hypothesis. Specifically, the positive link between institutional confidence and civic honesty was reversed among those who strongly endorse PDO. These findings suggest that individual‐level variation in the link between confidence in institutions and civic honesty partly reflects broader beliefs about authorities. We discuss implications of this interaction and outline directions for future research.

## 1 Introduction

Civic honesty refers to norms emphasising the importance of moral conduct in the context of public goods. These norms tend to be shared across contexts (Cohn et al. 2019) because they promote trust, cooperation and collective responsibility, serving as a foundation for functioning societies. Deviating from such norms undermines social cohesion, depletes public resources and affects community development. Hence, understanding the circumstances under which individuals are more likely to reject civic norms is crucial for designing effective public policy and civic education programmes.

Confidence in state institutions such as the government, the civil service and the police has been linked to a stronger endorsement of civic honesty (Letki 2006). However, emerging research reveals that in some circumstances, individuals with favourable views of institutions may paradoxically endorse lower standards of civic honesty (Chan et al. 2017; Travaglino et al. 2024). These findings highlight the need to investigate the psychological factors that might alter the relationship between institutional confidence and civic honesty.

The objective of this article is to explore one such factor. We draw on previous research on individuals' power‐distance orientation (PDO)—a cultural orientation that refers to the acceptance of power differentials in society—to test this construct's role in moderating the linkage between individuals' confidence in institutions and civic honesty (for a review on the effects of PDO at the cultural and individual level, see Winterich and Zhang 2014). Prior research indicates that power distance predicts both the tolerance and prevalence of corruption across societies (Boateng et al. 2024), potentially due to a lower propensity to challenge authorities (Hofstede et al. 2010). Notably, individuals endorsing a stronger PDO tend to perceive those at the top of the hierarchy as more entitled to privilege, encouraging relationships of patronage and clientelism with them (Davis and Ruhe 2003; García 2014). We hypothesised that expressing confidence in institutional authorities when such authorities are perceived as distant and potentially more likely to engage in corrupt practices may be associated with stronger civic dishonesty.

## 2 Civic Honesty, Confidence in Institutions and Power‐Distance Orientation

Civic honesty involves promoting the public good over personal gains (Letki 2006). Societies prosper when individuals internalise such norms (Letki 2006), and the endorsement of civic honesty may prevent citizens from engaging in tax avoidance or cheating on social benefits (Letki 2006; Torgler and Schneider 2007). A crucial driver of civic honesty is individuals' views of institutional authorities (Kubbe 2013; Letki 2006). According to legitimacy theory, confidence in such authorities reflects the extent to which individuals legitimise them (Tyler 1997). Confidence in institutions, in turn, enhances compliance with civic honesty because institutions are perceived as more likely to act in the public interest. Conversely, lower confidence in institutions can foster lenient attitudes towards corruption and unethical behaviour (Morris and Klesner 2010).

However, research shows substantial heterogeneity across contexts in this association (Kubbe 2013). An analysis of 108 countries found a positive link between confidence in parliament and tax morale in 73 such countries. In others, the relationship was null or negative (Chan et al. 2017). Additionally, Travaglino et al. (2024) found that in countries with more extreme levels of organised criminal activity, individuals' confidence in institutions is linked to lower endorsement of civic honesty.

Such variability across contexts raises the question of whether, for some individuals, confidence in institutions may paradoxically be associated with a rejection of civic norms. The present research examined the moderating role of PDO. PDO is particularly relevant because it shapes how individuals view their relationship with authority (Travaglino and Moon 2023).

In its original conceptualisation, PDO refers to a country‐level characteristic entailing a relationship of either interdependency or dependency between individuals and authorities (Hofstede et al. 2010). In low power‐distance societies, authority is marked by openness to dialogue, fostering interdependence. In contrast, high power‐distance societies normalise power disparities, discouraging dissent (Hofstede et al. 2010). Higher power‐distance societies also display greater tolerance towards the corruption of those in power (Boateng et al. 2024).

Beyond the country level, individuals differ in their PDO (Daniels and Greguras 2014; Winterich and Zhang 2014). High‐PDO individuals perceive hierarchies as deeply entrenched (Travaglino and Moon 2023). Previous work suggests that high PDO may render individuals more likely to view authorities as inclined to corruption and entitled to privileges (Davis and Ruhe 2003). High‐PDO individuals display a lower propensity to challenge superiors by reporting unethical behaviour in organisations (Daniels and Greguras 2014). Moreover, individuals who accept that power is concentrated at the top also become more accepting of patronage relationships with authorities as a strategy to obtain favours and resources (García 2014).

When individuals express confidence in—and thereby legitimise—institutional authorities while at the same time viewing such authorities as distant and potentially more inclined to corruption, they may become more likely to align their behaviour with the expected norms of those in power. Thus, in the present study, we tested the hypothesis that stronger confidence in institutions, when combined with higher PDO, may predict the rejection of civic honesty norms. We tested our hypothesis in a sample of participants from eight countries.

## 3 Methods

### 3.1 Participants

Data were collected via Qualtrics by a panel company in eight countries (*N* = 2088) from different world regions. Sample characteristics are summarised in Table 1. All data, script and Supporting Information for this study can be found at https://osf.io/c7b6z/?view_only=4319462eeffa48b68444ea3b60d279a8.

**TABLE 1:** Sample size, age mean and standard deviation and gender composition in the eight countries surveyed.

	*N*	*M*_age_	SD_age_	% Women
U.S.	277	46.18	16.65	49%
U.K.	257	47.88	15.83	49%
Italy	258	48.63	16.09	53%
South Korea	256	43.05	14.61	65%
Japan	264	50.57	16.46	58%
Germany	259	51.59	15.39	49%
Chile	260	41.23	13.89	55%
Colombia	257	39.35	12.86	53%

### 3.2 Measures

Participants completed a survey that included our three focal measures in a randomised order. To measure civic honesty, we asked respondents to consider the justifiability of four actions (Harding et al. 1986), ‘claiming state benefits to which you are not entitled’, ‘avoiding a fare on public transport’, ‘cheating on taxes if you have a chance’ and ‘someone accepting a bribe in the course of their duties’ (1 = *never justifiable*, 10 = *always justifiable*, Cronbach's *α* = 0.88). Items were reversed: higher scores indicate stronger endorsement of civic honesty.

Three items measured participants' PDO (drawn from Winterich and Zhang 2014): ‘People in higher positions should make most decisions without consulting people in lower positions’, ‘People in lower positions should not disagree with decisions by people in higher positions’, ‘People in higher positions should avoid social interaction with people in lower position’ (1 = *strongly disagree* to 7 = *strongly agree; α* = 0.85).

Confidence in institutions was measured by asking respondents to indicate their confidence in six major domestic institutions: the police, parliament, civil service, government, political parties and the justice system/courts (1 = *not at all*, 7 = *extremely high*; *α* = 0.90).

In our model, we controlled for age and gender because previous research has shown that they impact attitudes towards corruption (Letki 2006). We also accounted for individuals' subjective socioeconomic status (SSS) and political orientation. To measure SSS, we asked respondents to place themselves on a ladder representing people who are best and worst off in society. Finally, we measured political orientation by asking respondents how they would describe themselves from 0 = *I am a left‐winger* to 10 = *I am a right‐winger*.

### 3.3 Analytical Strategy

In the analyses, we included country fixed effects to account for participants' clustering in nations (McNeish and Kelley 2019). This method removes cross‐country variability and is particularly suited for analysing clustered data with a small number of clusters. The inclusion of fixed effects means that results can be interpreted as within‐country averages. The predicted interaction between PDO and confidence in institutions was tested using latent variables. We employed a product‐indicator approach with residual centering. Residual centering yields results comparable to other techniques for testing latent interactions (e.g., mean centering or double mean centering) while imposing fewer restrictions on the model (Schoemann and Jorgensen 2021).

## 4 Results

Table 2 summarises correlations among variables, means and standard deviations for the whole sample. Before testing our hypothesis, we examined the invariance of the measures. We tested whether construct structures (i.e., configural invariance), factor loadings (i.e., metric invariance) and intercepts (i.e., scalar invariance) could be constrained across countries. Tests were conducted using the ΔCFI < 0.01 criterion (Chen 2007).

**TABLE 2:** Means, standard deviations and correlations for study variables.

Variable	*M*	SD	1	2	3	4	5	6
1. Civic honesty	8.71	1.89						
2. Confidence in institutions	3.39	1.38	−0.12**					
3. Power distance	2.17	1.36	−0.38**	0.23**				
4. Age	46.08	15.81	0.23**	0.13**	−0.04			
5. Gender	1.47	0.51	−0.01	−0.04	−0.13**	−0.05*		
6. SSS	5.81	1.76	0.05*	−0.21**	−0.07**	−0.05*	0.00	
7. Political or.	5.51	2.31	−0.02	0.07**	0.20**	0.05*	−0.05*	−0.14**

*Note:* Correlation between variables for the entire sample (*N* = 2088).

Abbreviation: SSS, subjective socioeconomic status.

*p < 0.05, **p < 0.01.

All measures achieved at least partial scalar invariance (see Supporting Information for details). To test our moderation hypothesis, we employed a model that included latent (PDO, confidence and civic honesty) and observed variables (demographics). Robust standard errors were used to account for normality violations. The model had adequate fit, *χ* ^2^ (190, *N* = 2058) = 1843.959, *p* < 0.001, CFI = 0.90, RMSEA = 0.07, SRMR = 0.06.

We found a significant main effect of PDO (*b* = −0.561, SE = 0.046, 95% CI [−0.652, −0.470], *p* < 0.001), suggesting that individuals who tend to see hierarchies as fixed and accept the large distance between the powerful and the powerless are more likely to reject civic honesty. There was no significant main effect of confidence in institutions (*b =* −0.033, SE = 0.031, 95% CI [−0.093, 0.028], *p* = 0.293). The hypothesised interaction between PDO and confidence was significant (*b* = −0.260, SE = 0.047, 95% CI [−0.352, −0.169], *p* < 0.001). Among covariates, only age had a significant relationship with civic honesty (*b* = 0.02, SE = 0.00, 95% CI [0.014, 0.021], *p* < 0.001).

**FIGURE 1:**
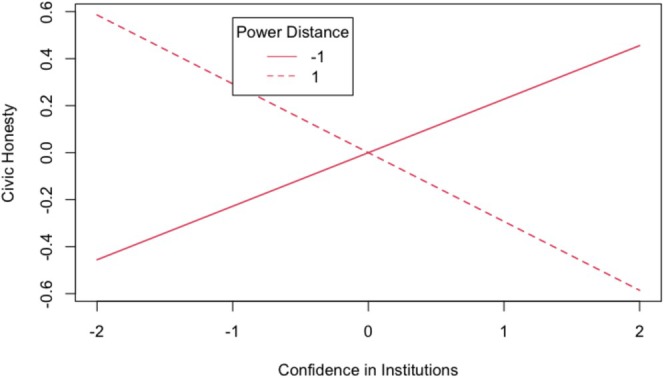
Simple slope analysis of the relationship between confidence in institutions and civic honesty at ±1 SD values of power‐distance orientation. Gender, age, political orientation and subjective socioeconomic status were covariates in the model.

To probe the latent interaction, we conducted a simple slope analysis of confidence at different levels of PDO (±1 SD; see Figure 1). In line with our hypothesis, we found a significant positive association between confidence and civic honesty among individuals with lower PDO, *b* = 0.228, SE = 0.054, 95% CI [0.120, 0.336], *p* < 0.001. Conversely, confidence was negatively associated with civic honesty when PDO was higher, *b* = −0.293, SE = 0.071, 95% CI [−0.435, −0.151], *p* < 0.001. Individuals who expressed confidence in institutions but at the same time endorsed high power differentials also regarded civic dishonesty as more justifiable.

## 5 Discussion

Prior evidence generally indicates a positive association between institutional confidence and civic honesty (Chan et al. 2017; Letki 2006). However, in some contexts, this association has been found to be null or negative (Chan et al. 2017; Kubbe 2013; Travaglino et al. 2024). This study investigated an individual‐level factor that could moderate the association between confidence in institutions and civic honesty.

We focused on PDO (Hofstede et al. 2010). Drawing on past research on PDO and corruption (Boateng et al. 2024), we anticipated that confidence in institutions and the political system, combined with higher PDO, would be linked to the rejection of civic honesty norms.

Our results supported this hypothesis. At lower levels of PDO, expressing confidence in institutions was linked to a stronger endorsement of civic honesty norms. This finding replicates prior evidence (Letki 2006) and is in line with legitimacy theory (Tyler 1997), which emphasises the importance of individuals' confidence in institutions in driving their respect for civic norms. Conversely, and consistent with our reasoning, the relationship between confidence and civic honesty was negative at higher levels of PDO. This finding clarifies past work on the paradoxical effects of trust (Chan et al. 2017; Travaglino et al. 2024) by showing that confidence in institutions is associated with a lower endorsement of civic honesty when individuals hold the view that large power differentials are a necessary characteristic of society.

The belief that power differentials are both necessary and unchangeable has been associated with a greater prevalence and tolerance of corruption (Boateng et al. 2024). When high PDO is coupled with strong confidence in institutions, individuals may become more accepting of the authority of those in power, regardless of how this power was achieved. This acceptance can foster the internalisation of the authority's norms (Tyler 1997), even though these norms promote civic dishonesty. These findings provide new insights into how confidence in institutions can backfire in some circumstances. Future research should directly examine the internalisation of norms to determine whether they mediate the interactive effect of institutional confidence and PDO on the endorsement of civic honesty.

Another important direction for future research concerns the generalisability of the findings across contexts. Because we employed fixed effects to account for cross‐country variability, the results cannot be generalised beyond the countries included in the model. Future research should include a broader range of countries and adopt hierarchical linear models to explore how macro‐level factors—such as institutional quality (Acemoglu and Robinson 2012), country‐level power distance and economic or political conditions—shape the relationship between PDO, institutional trust and civic honesty. Finally, it is also crucial to account for socio‐cultural and political differences across contexts, including variations in how individuals interpret scales and the influence of ethnicity in multicultural samples.

## 6 Conclusions

Civic honesty is crucial for the prosperity of society. Typically, individuals' endorsement of civic honesty is predicted by positive views of institutions and the political system. However, there are circumstances where expressing confidence in institutions may be linked to lower honesty. Results indicate that when individuals express confidence in institutions while also perceiving power differentials as entrenched in society, they are more likely to justify dishonesty and corruption. Our findings underscore the importance of examining unexplored factors, such as PDO, to further understand the complex relationship between individuals' views of institutions and civic honesty. Additionally, these results have significant implications for policymakers, highlighting the need to address perceptions of power imbalances and promote inclusivity to foster civic honesty and reduce the justification of corrupt behaviours.

## Ethics Statement

This study was conducted in accordance with the Institutional Review Board and the 1964 Helsinki Declaration. All participants provided informed consent prior to their involvement, and the research was approved by the Ethics Review Board Royal Holloway, University of London (Code no. 3745‐2023).

## Conflicts of Interest

The authors declare no conflicts of interest.

## Supporting information


Data S1.

## Data Availability

Data and materials can be found at https://osf.io/c7b6z/?view_only=4319462eeffa48b68444ea3b60d279a8.
