# Supplementary material for: When Confidence in Institutions Backfires: Power‐Distance Orientation Moderates the Relationship Between Institutional Trust and Civic Honesty Across Eight Countries
Source: Int J Psychol. 2025 May 28;60(4):e70059. doi: 10.1002/ijop.70059 (PMC12120376; doi:10.1002/ijop.70059)
Supplement: Supplementary file 1 — Data S1. [file IJOP-60-e70059-s001.docx]

Supplementary Material for the article When Confidence in Institutions Backfires: Power Distance Orientation Moderates the Relationship Between Institutional Trust and Civic Honesty Across Eight Countries

1. **Supplementary Demographics**

Table 1. Mean and Standard Deviation (in parentheses) for Political Orientation and Socioeconomic Status Across the 8 Countries

|  | U.S. | U.K. | Italy | South Korea | Japan | Germany | Chile | Colombia |
| --- | --- | --- | --- | --- | --- | --- | --- | --- |
| *N* | 277 | 257 | 258 | 256 | 264 | 259 | 260 | 257 |
| Political Orientation | 5.48 (2.69) | 5.09 (2.07) | 5.40 (2.65) | 5.73 (1.93) | 5.66 (1.71) | 5.08 (1.78) | 5.92 (2.71) | 5.72 (2.57) |
| Socio-economic Status | 5.96 (1.93) | 5.81 (1.86) | 5.54 (1.62) | 6.07 (1.77) | 6.22 (1.79) | 6.03 (1.75) | 5.3 (1.54) | 5.55 (1.56) |

1. **Supplementary information on the analysis**

We first ran invariance tests for our three focal measures: civic honesty, power distance orientation and confidence in institutions. The aim of these tests is to check whether we are measuring the same constructs across countries.

To assess invariance for the three focal constructs, we began by specifying their measurement models and evaluating whether these models were consistent across countries, known as configural invariance. Following this, we tested for metric invariance by examining whether factor loadings could be constrained to remain equal across countries. Finally, scalar invariance was tested by determining if intercepts could also be constrained across groups. For cases where full invariance was not supported, partial invariance was explored by identifying the specific loadings or intercepts that contributed to misfit and allowing them to vary across countries. Invariance was assessed using changes in CFI, with a threshold of ≤ .01 (Cheung & Rensvold, 2002).

**2.1 Invariance Test: Civic Honesty**

Table 2. Invariance test (Civic Honesty)

|  | **chisq** | **df** | **pvalue** | **rmsea** | **cfi** | **tli** | **srmr** |
| --- | --- | --- | --- | --- | --- | --- | --- |
| configural | 112.974† | 16 | .000 | .152 | .980† | .941 | .020† |
| metric | 179.850 | 37 | .000 | .122† | .971 | .962† | .053 |
| scalar | 364.581 | 58 | .000 | .142 | .938 | .948 | .071 |

The measure of civic honesty had an RMSEA = .152 and CFI =. .98. Constraining loadings to be equal across groups minimally worsened the model fit (CFI = .97, ΔCFI = .01). However, the model did not achieve full scalar invariance, ΔCFI = .03. Releasing the intercept of one of the items (“Avoiding a fare on public transport”) enabled the measure to achieve partial scalar invariance, ΔCFI = .01.

Table 3. Invariance test releasing intercept of item 2 (Civic Honesty)

|  | **chisq** | **df** | **pvalue** | **rmsea** | **cfi** | **tli** | **srmr** |
| --- | --- | --- | --- | --- | --- | --- | --- |
| configural | 112.974† | 16 | .000 | .152 | .980† | .941 | .020† |
| metric | 179.850 | 37 | .000 | .122 | .971 | .962 | .053 |
| scalar | 242.651 | 51 | .000 | .120† | .961 | .963† | .059 |

**2.2 Invariance Test: Power-Distance Orientation**

The measure of power-distance orientation’s configural model was just-identified (RMSEA  =  0, CFI  =  1). Constraining loadings to be equal across groups (metric invariance) only minimally worsened the model fit (CFI = .99, ΔCFI = .01). Likewise, constraining items’ intercepts to be equal across groups minimally worsened the model fit (CFI = .98, ΔCFI = .01). Thus, scalar invariance was achieved for power-distance orientation.

Table 4. Invariance Test (Power Distance Orientation)

|  | **chisq** | **df** | **pvalue** | **rmsea** | **cfi** | **tli** | **srmr** |
| --- | --- | --- | --- | --- | --- | --- | --- |
| configural | .000† |  | NA | .000† | 1.000† | 1.000† | .000† |
| metric | 17.620 | 14 | .225 | .031 | 0.999 | 0.998 | .025 |
| scalar | 61.937 | 28 | .000 | .068 | .987 | .989 | .039 |

**2.3 Invariance Test: Confidence in Institutions**

Finally, the configural model for the measure of confidence in institutions did not initially achieve a good fit, RMSEA  =  .11, and configural invariance was not achieved, ΔCFI = .02

Table 5. Invariance Test (Confidence in Institutions)

|  | **chisq** | **df** | **pvalue** | **rmsea** | **cfi** | **tli** | **srmr** |
| --- | --- | --- | --- | --- | --- | --- | --- |
| configural | 159.700† | 40 | .000 | .107† | .986† | .959† | .020† |
| metric | 343.978 | 75 | .000 | .117 | .969 | .951 | .084 |
| scalar | 871.372 | 110 | .000 | .163 | .913 | .905 | .118 |

Table 6. Invariance test releasing intercept of items (Confidence in Institutions)

|  | **chisq** | **df** | **pvalue** | **rmsea** | **cfi** | **tli** | **srmr** |
| --- | --- | --- | --- | --- | --- | --- | --- |
| configural | 159.700† | 40 | .000 | .107† | .986† | .959† | .020† |
| metric | 249.323 | 54 | .000 | .118 | .978 | .951 | .056 |
| scalar | 330.303 | 61 | .000 | .130 | .969 | .940 | .072 |

After inspecting the modification indices, we allowed the residuals of the items to covary. The resulting model had a similar fit, CFI = 0.98, RMSEA = 0.11; however configural, ΔCFI = .01, and partial scalar invariance was achieved ΔCFI = .01.

**2.4 Interaction model with latent variables:**

We tested our main hypothesis (power distance orientation will moderate the relationship between confidence in institutions and civic honesty) using a structural equation model with latent and observed variables. The structural equation model included the focal test of a latent interaction using a product-indicator approach with residual centering. To address the issue of participants clustering in different countries, our model includes country-level fixed effects. The main results of the model are displayed in Table 7. In the main text of this article, we discuss the results of the interaction. Overall, we found a negative main effect of power-distance orientation on civic honesty, a positive effect of age (older people report higher civic honesty) and negative main effects of being from either the U.S. or Germany (lower civic honesty compared to Italy). On the contrary, people living in Japan and Colombia reported higher civic honesty than the referent group.

Table 7. Latent Regression Model for the Moderation Between Power-Distance and Confidence in Institutions over Civic Honesty

|  | Estimate | Std.Err | z-value | P(>\|z\|) | Stand. Estim. |
| --- | --- | --- | --- | --- | --- |
| Confidence in Institutions | -0.033 | 0.031 | -1.052 | 0.293 | -0.026 |
| Power-distance | -0.561 | 0.046 | -12.111 | **0.000** | -0.454 |
| Confidence *Power-distance | -0.260 | 0.047 | -5.591 | **0.000** | -0.211 |
| Gender | -0.010 | 0.049 | -0.208 | 0.835 | -0.004 |
| Age | 0.018 | 0.002 | 10.521 | **0.000** | 0.224 |
| Socioeconomic Status | 0.028 | 0.016 | 1.750 | 0.080 | 0.039 |
| Political Orientation | 0.009 | 0.011 | 0.782 | 0.434 | 0.017 |
| Country UK | -0.120 | 0.095 | -1.263 | 0.207 | -0.032 |
| Country US | -0.391 | 0.101 | -3.865 | **0.000** | -0.106 |
| Country GE | -0.579 | 0.111 | -5.240 | **0.000** | -0.154 |
| Country JA | 0.240 | 0.090 | 2.671 | **0.008** | 0.065 |
| Country KO | 0.057 | 0.097 | 0.587 | 0.557 | 0.015 |
| Country CO | 0.228 | 0.088 | 2.588 | **0.010** | 0.061 |
| Country CL | 0.066 | 0.095 | 0.699 | 0.485 | 0.018 |

Note. Countries, UK = United Kingdom, US = United States, GE = Germany, JA = Japan, KO = South Korea, CO = Colombia, CL = Chile (Referent country = Italy). Bold indicates significance.
